# Trends in the Use of Stereotactic Body Radiotherapy for Treatment of Prostate Cancer in the United States

**DOI:** 10.1001/jamanetworkopen.2019.20471

**Published:** 2020-02-05

**Authors:** Sean S. Mahase, Debra D’Angelo, Josephine Kang, Jim C. Hu, Christopher E. Barbieri, Himanshu Nagar

**Affiliations:** 1 Department of Radiation Oncology, Weill Cornell Medicine, New York, New York; 2 Division of Biostatistics and Epidemiology, Department of Healthcare Policy and Research, Weill Cornell Medicine, New York, New York; 3 Department of Urology, Weill Cornell Medicine, New York, New York

## Abstract

**Importance:**

Stereotactic body radiotherapy is a hypofractionated, cost-effective treatment option for localized prostate cancer.

**Objective:**

To characterize US national trends and the clinical and socioeconomic factors associated with the use of stereotactic body radiotherapy in prostate cancer.

**Design, Setting, and Participants:**

This retrospective cohort study used data collected by the National Cancer Database to assess the clinical and socioeconomic factors among 106 926 men diagnosed as having prostate cancer from 2010 to 2015 who underwent definitive radiotherapy and the trends in the use of this therapy. The initial analysis was performed between January and February 2018, with final updates performed August 2019.

**Exposure:**

Stereotactic body radiotherapy, defined as 5 fractions of radiotherapy.

**Main Outcomes and Measures:**

Temporal trends and clinical and sociodemographic factors associated with stereotactic body radiotherapy use.

**Results:**

In total, 106 926 patients diagnosed as having localized prostate cancer between 2010 and 2015 and receiving definitive radiotherapy were identified. White patients composed 77.3% of this cohort, whereas black patients composed 18.7%. Government-issued insurance was used by 61.2% of patients. More than 80% of patients had a Charlson-Deyo Comorbidity Index score of 0 (range, 0 to ≥3, with lower numbers indicating fewer comorbidities). In the study population, 25.7% had low-risk disease; 26.3%, favorable intermediate-risk disease; 23.3%, unfavorable intermediate-risk disease; and 24.7%, high-risk disease. The proportion of patients who underwent radiotherapy and received stereotactic body radiotherapy (a total of 5395 patients) increased from 3.1% in 2010 to 7.2% in 2015 (odds ratio, 0.36; 95% CI, 0.33-0.40; *P* < .001). Among the entire cohort, patients received a median dose of 36.25 Gy (range, 30.00-50.00 Gy). Androgen deprivation therapy use increased significantly as disease risk level increased among all patients receiving radiotherapy (9.5% with low risk to 76.6% with high risk; *P* = .02) and among those receiving stereotactic body radiotherapy (4.1% with low risk to 33.2% with high risk; *P* = .04) or not receiving stereotactic body radiotherapy (9.9% with low risk to 77.6% with high risk; *P* = .04). Patients treated at an academic center, living in an urban area, or possessing higher incomes and those who were healthier, white individuals, or were diagnosed as having lower-risk prostate cancer had higher odds of receiving stereotactic body radiotherapy.

**Conclusions and Relevance:**

This study found that stereotactic body radiotherapy use in prostate cancer more than doubled from 2010 to 2015 but accounted for less than 10% of all patients undergoing radiotherapy. Androgen deprivation therapy use increased with disease risk among patients overall, regardless of receiving stereotactic body radiotherapy. Socioeconomic and clinical determinants of stereotactic body radiotherapy included risk category, Charlson-Deyo Comorbidity Index score, facility type and location, income, race/ethnicity, and year of diagnosis. These results are hypothesis generating; further studies evaluating potential disparities in stereotactic body radiotherapy use in localized prostate cancer are warranted.

## Key Points

**Question:**

Does an association exist between clinical or socioeconomic factors and selecting stereotactic body radiosurgery to treat prostate cancer in the United States?

**Findings:**

In a cohort study using data from the National Cancer Database, the proportion of 106 926 patients with prostate cancer undergoing stereotactic body radiotherapy significantly increased from 3.1% in 2010 to 7.2% in 2015. Being a white individual, treated in an academic center, living in an urban locale, and having higher income, fewer comorbidities, and lower-risk prostate cancer were associated with receiving stereotactic body radiotherapy.

**Meaning:**

These results suggest that definitively treating prostate cancer with stereotactic body radiotherapy is increasing, but several clinical factors and socioeconomic disparities may influence its implementation.

## Teaser

This cohort study uses data from the National Cancer Database to analyze trends in prostate cancer treatment to assess whether an association exists between clinical or socioeconomic factors and selecting stereotactic body radiosurgery for treatment of US men with prostate cancer.

## Introduction

Prostate cancer is the most common malignant neoplasm in men, with more than 164 000 new cases diagnosed in 2018.^1^ With the advent and availability of prostate-specific antigen (PSA) testing enabling earlier detection of prostate cancer, most men are diagnosed as having localized disease. Prostate cancer is stratified as low-, intermediate-, and high-risk disease based on tumor stage, PSA level at diagnosis, and Gleason score.^2^ Treatment options for localized prostate cancer include active surveillance (reserved for low-risk individuals), radical prostatectomy with possible pelvic lymph node dissection, or radiation therapy (RT) entailing brachytherapy, external beam radiation therapy (EBRT), or both.

Fractionated EBRT typically involves a 9- to 10-week treatment with 1.8 to 2.0 Gy delivered daily. Technological advancements in parallel with improvements in treatment software enable more conformal EBRT planning, resulting in higher cumulative dose delivery while minimizing toxic effects.^3,4^ These innovations have promoted studies showing comparable toxicity profiles and noninferior outcomes between conventional EBRT given during 9 weeks to moderately hypofractionated regimens administered for 4 to 6 weeks.^5,6,7^ Furthermore, the radiobiological rationale that a slow proliferation rate of prostate cancer is reflected by an α:β ratio of 2:3, similar to that of adjacent toxic effect–limiting organs,^8^ suggests that extremely hypofractionated radiation regimens will provide comparable cancer control rates without increasing toxic effects.

Stereotactic body radiotherapy (SBRT) is an extremely hypofractionated regimen of 7 to 10 Gy per fraction delivered using stereotactic or image-guided intensity-modulated RT techniques. Stereotactic body radiotherapy is most commonly completed in 5 total fractions, enabling patients to finish their treatment in fewer visits, thereby increasing convenience while lowering health care costs. Numerous studies have validated 5-fraction prostate SBRT as a cost-effective, noninvasive treatment achieving outcomes equivalent to conventionally fractionated EBRT or to surgery without compromising safety.^9,10,11,12,13^ These findings have culminated in National Comprehensive Cancer Network guidelines supporting 5-fraction prostate SBRT use at facilities with appropriate technology and physics and clinical expertise.^2^ The present study aims to characterize national trends in SBRT use.

## Methods

### Data Source

The National Cancer Database (NCDB) is an oncology-focused national database established by the American College of Surgeons and the Commission on Cancer of the American College of Surgeons. The NCDB tabulates longitudinal data from more than 70% of all new cancer diagnoses on an annual basis, encompassing more than 1500 hospital across all 50 US states.^14^ The collected data include cancer characteristics, primary and adjuvant management, and long-term outcomes, as well as patient demographic information, such as age, sex, race/ethnicity, educational level, income, and insurance status.^15^ The present study follows the Strengthening the Reporting of Observational Studies in Epidemiology (STROBE) reporting guideline for cohort studies. Because the study used deidentified data from the NCDB database, the requirement for formal institutional review and the need for informed patient consent were waived, consistent with the policies of Weill Cornell Medicine, New York, New York.

### Study Population

The NCDB was queried to identify patients diagnosed as having localized prostate cancer from 2010 to 2015 who underwent definitive RT. Stereotactic body radiotherapy was defined as patients undergoing treatment with 5 fractions of EBRT. Prostate cancer diagnoses were made via biopsy of the primary site. Patients were excluded from analysis if they lacked sufficient clinical data to assess their disease risk stratification, received a diagnosed at the reporting facility but had their treatment decision made elsewhere, or underwent surgery or chemotherapy as part of their initial treatment. They were also excluded if their RT total dose appeared erroneous (eg, SBRT<30 Gy or >50 Gy). Patients meeting all of the following criteria were classified as having low-risk disease: American Joint Committee on Cancer (AJCC) tumor category cT1a to cT2a, Gleason score of 6 (with lower numbers indicating cells that are well differentiated and look similar to healthy cells), and PSA level less than 10 ng/mL (to convert PSA level to micrograms per liter, multiply by 1). Patients with tumor category cT2b to cT2c or Gleason score of 3 + 4 or 4 + 3 or PSA level of 10 to 20 ng/mL were classified as having intermediate-risk disease. The percentage of biopsy core results that were positive was not available in the data set for analysis. Patients with only 1 intermediate-risk criterion were defined as having favorable intermediate-risk disease, whereas patients with a Gleason score of 4 + 3 or with at least 2 intermediate-risk characteristics were classified as having unfavorable intermediate-risk disease. Patients with tumor category cT3a to cT4 or Gleason score of 8 or higher or PSA level higher than 20 ng/mL were defined as having high-risk disease.^2^ Other variables of interest queried for analysis included the type of facility, geographic facility location, race/ethnicity, insurance status, income, metropolitan vs rural locale, and Charlson-Deyo comorbidity index score (range, 0 to ≥3, with lower numbers indicating fewer comorbidities).

### Statistical Analysis

Descriptive statistics for factors of interest were reported as frequency (percentage). The Cochran-Armitage test was used to identify significant trends in the use of SBRT with time and by risk category. Multivariable logistic regression was performed to examine clinical and sociodemographic factors associated with SBRT use. All tests were 2-sided and considered significant at an α level of .05. All analyses were performed using SAS software, version 9.4 (SAS Institute Inc). Initial analyses were performed between January and February 2018, with final updates performed August 2019.

## Results

Demographic and clinical characteristics are given in Table 1. Overall, 106 926 patients diagnosed as having prostate cancer from 2010 to 2015 underwent definitive RT. A flow diagram outlining the cohort selection is shown in the eFigure in the Supplement. White individuals composed 77.3% of this cohort, whereas black persons composed 18.7% of the study population. Government-issued insurance, including Medicare and Medicaid, was used by 61.2% of patients, whereas 35.9% used private insurance. The majority of patients underwent treatment at a comprehensive community cancer program (46.9%) or an academic institution (32.5%). More than 80% of the cohort had a Charlson-Deyo comorbidity index score of 0. The study population comprised patients with low-risk (25.7%), favorable intermediate-risk (26.3%), unfavorable intermediate-risk (23.3%), and high-risk (24.7%) disease.

**Table 1.** Demographic and Clinical Characteristics of 106 926 Patients Diagnosed as Having Prostate Adenocarcinoma From 2010 to 2015 Who Underwent Definitive Radiation Therapy

Patient Characteristic	Frequency (%)
Year of diagnosis	
2010	20 862 (19.5)
2011	20 633 (19.3)
2012	15 444 (14.4)
2013	15 505 (14.5)
2014	16 860 (15.8)
2015	17 622 (16.5)
Race/ethnicity	
White	82 697 (77.3)
Black	20 032 (18.7)
Other	3095 (2.9)
Unknown	1102 (1.0)
Primary payer	
Private insurance	38 382 (35.9)
Medicaid, Medicare, or other government insurance	65 393 (61.2)
Uninsured	1646 (1.5)
Unknown	1505 (1.4)
Median annual income, $	
<38 000	18 725 (17.5)
38 000-47 999	24 018 (22.5)
48 000-62 999	28 119 (26.3)
≥63 000	35 752 (33.4)
Missing	312 (0.3)
Facility type	
Community cancer program	11 040 (10.3)
Comprehensive community cancer program	50 108 (46.9)
Academic or research program	34 795 (32.5)
Integrated network cancer program	10 973 (10.3)
Missing	10 (0.01)
Facility location	
New England	7557 (7.1)
Mid-Atlantic	17 716 (16.6)
South Atlantic	27 365 (25.6)
Central	
East North	19 495 (18.2)
East South	7510 (7.0)
West North	7430 (7.0)
West South	4300 (4.0)
Mountain	3631 (3.4)
Pacific	11 912 (11.1)
Missing	10 (0.01)
Location type	
Metropolitan	86 603 (81.0)
Urban	15 574 (14.6)
Rural	2236 (2.1)
Missing	2513 (2.4)
AJCC T category	
cT1a	163 (0.2)
cT1b	152 (0.1)
cT1c	77 219 (72.2)
cT2a	12 165 (11.4)
cT2b	6637 (6.2)
cT2c	7217 (6.8)
cT3a	1850 (1.7)
cT3b	1284 (1.2)
cT4	239 (0.2)
AJCC N category	
cN0	104 938 (98.1)
cNx	1988 (1.9)
PSA, ng/mL	
<10	77 555 (72.5)
10-20	18 159 (17.0)
>20	11 212 (10.5)
Gleason score	
3 + 3	35 004 (32.7)
3 + 4	33 841 (31.7)
3 + 5	862 (0.8)
4 + 3	16 834 (15.7)
4 + 4	11 234 (10.5)
4 + 5	6471 (6.1)
5 + 3	264 (0.3)
5 + 4	1733 (1.6)
5 + 5	683 (0.6)
Charlson-Deyo Comorbidity Index score	
0	89 313 (83.5)
1	14 384 (13.5)
2	2398 (2.2)
≥3	831 (0.8)
Risk stratification	
Low	27 500 (25.7)
Intermediate	
Favorable	28 085 (26.3)
Unfavorable	24 960 (23.3)
High	26 381 (24.7)

Abbreviations: AJCC, American Joint Committee on Cancer; PSA, prostate-specific antigen.

SI conversion factor: To convert PSA levels to micrograms per liter, multiply by 1.

Of 5395 patients with prostate cancer undergoing SBRT, the proportion increased from 3.1% in 2010 to 7.2% in 2015 (*P* < .001) (Table 2). This trend persisted across all risk groups, including low risk (3.8% in 2010 to 12.2% in 2015; *P* < .001), favorable intermediate risk (3.6% in 2010 to 10.2% in 2015; *P* < .001), unfavorable intermediate risk (2.9% in 2010 to 6.8% in 2015; *P* < .001), and high risk (1.6% in 2010 to 2.0% in 2015; *P* = .046). Conversely, the number of patients receiving EBRT steadily decreased from 20 218 in 2010 to 16 346 in 2015. Stratifying by risk indicated that the annual number of patients classified as having high-risk disease (4453 in 2010 to 4981 in 2015) and unfavorable intermediate-risk disease (4165 in 2010 to 4476 in 2015) treated with EBRT remained relatively constant; however, there was a consistent decrease in patients with favorable intermediate-risk disease (5069 in 2010 to 4284 in 2015) and low-risk disease (6531 in 2010 to 2605 in 2015) treated annually during this time. The entire cohort receiving SBRT, and on stratification by risk group, received a median dose of 36.25 Gy (range, 30.00-50.00 Gy) (Table 3). Among all patients receiving definitive RT, 39.1% received androgen deprivation therapy (ADT), and 13.0% of patients undergoing SBRT received ADT (Table 4). The use of ADT in patients treated with definitive RT was higher in higher-risk groups (low risk, 9.5%; favorable intermediate risk, 24.7%; unfavorable intermediate risk, 48.2%; and high risk, 76.6%; *P* = .02). Androgen deprivation therapy use was also increased with higher-risk disease in those receiving SBRT (low risk, 4.1%; favorable intermediate risk, 10.7%; unfavorable intermediate risk, 20.3%; and high risk, 33.2%; *P* = .04) or not receiving SBRT (low risk, 9.9%; favorable intermediate risk, 25.7%; unfavorable intermediate risk, 49.6%; and high risk 77.6%; *P* = .04).

**Table 2.** Trend of SBRT vs Other RT by Risk Group and Year (2010-2015)

Risk Category	RT Group	Year of Diagnosis, No. (%)						*P* Value^a^
		2010	2011	2012	2013	2014	2015	
Low	SBRT	258 (3.8)	273 (4.3)	270 (6.5)	327 (8.8)	310 (9.0)	363 (12.2)	<.001
	Other	6531 (96.2)	6134 (95.7)	3900 (93.5)	3393 (91.2)	3136 (91.0)	2605 (87.8)	
Intermediate								
Favorable	SBRT	190 (3.6)	246 (4.6)	231 (5.7)	294 (7.0)	348 (7.8)	486 (10.2)	<.001
	Other	5069 (96.4)	5116 (95.4)	3814 (94.3)	3919 (93.0)	4088 (92.2)	4284 (89.8)	
Unfavorable	SBRT	124 (2.9)	141 (3.3)	142 (4.0)	235 (6.3)	257 (6.0)	327 (6.8)	<.001
	Other	4165 (97.1)	4139 (96.7)	3420 (96.0)	3524 (93.8)	4010 (94.0)	4476 (93.2)	
High	SBRT	72 (1.6)	91 (2.0)	90 (2.5)	99 (2.6)	121 (2.6)	100 (2.0)	.046
	Other	4453 (98.4)	4493 (98.0)	3577 (97.6)	3714 (97.4)	4590 (97.4)	4981 (98.0)	
All patients	SBRT	644 (3.1)	751 (3.6)	733 (4.8)	955 (6.2)	1036 (6.1)	1276 (7.2)	<.001
	Other	20 218 (96.9)	19 882 (96.4)	14 711 (95.3)	14 550 (93.8)	15 824 (93.9)	16 346 (92.8)	

Abbreviations: RT, radiation therapy; SBRT, stereotactic body radiation therapy.

^a^ Trend analyzed using the Cochran-Armitage test.

**Table 3.** Radiation Dose by Risk Group in Patients Receiving 5 Fractions of Definitive Radiotherapy

Risk Group	No.	Total Dose, Median (Range), Gy
Low	1801	36.25 (30.00-50.00)
Intermediate		
Favorable	1795	36.25 (30.00-50.00)
Unfavorable	1226	36.25 (30.00-50.00)
High	573	36.25 (32.50-45.00)

**Table 4.** Trend in Use of ADT by Risk Group and RT Status

RT Status	ADT Use	Risk Level, No. (%)				*P* Value^a^
		Low	Intermediate		High	
			Favorable	Unfavorable		
All	Yes	2607 (9.5)	6949 (24.7)	12 031 (48.2)	20 214 (76.6)	.02
	No	24 893 (90.5)	21 136 (75.3)	12 929 (51.8)	6167 (23.4)	
SBRT only	Yes	73 (4.1)	192 (10.7)	249 (20.3)	190 (33.2)	.04
	No	1728 (96.0)	1603 (89.3)	977 (79.7)	383 (66.8)	
Non-SBRT only	Yes	2534 (9.9)	6757 (25.7)	11 782 (49.6)	20 024 (77.6)	.04
	No	23 165 (90.1)	19 533 (74.3)	11 952 (50.4)	5784 (22.4)	

Abbreviations: ADT, androgen deprivation therapy; RT, radiation therapy; SBRT, stereotactic body radiation therapy.

^a^ Trend analyzed using the Cochran-Armitage test.

Multivariable analysis results are presented in Table 5. Patients treated outside of an academic institution were less likely to receive SBRT (community cancer center program: odds ratio [OR], 0.22; 95% CI, 0.18-0.25; *P* < .001; comprehensive community cancer center program: OR, 0.39; 95% CI; 0.36-0.41; *P* < .001; integrated network cancer program: OR, 0.46; 95% CI, 0.41-0.51; *P* < .001), and those who lived in an urban area were more likely than those in a metropolitan location to receive SBRT (OR, 1.27; 95% CI, 1.15-1.41; *P* < .001). Black patients (OR, 0.91; 95% CI, 0.84-0.99; *P* = .02) and other races/ethnicities had lower odds than white patients of receiving SBRT. Patients with more comorbidities had lower odds of receiving SBRT than those without comorbidity (eg, 1 comorbidity: OR, 0.93; 95% CI, 0.85-1.01; *P* < .001). Patients in New England (the reference) and the Pacific Coast (OR, 0.25; 95% CI, 0.20-0.31; *P* < .001) had lower odds of receiving SBRT than other regions in the United States. In addition, patients with higher median annual incomes had greater odds of receiving SBRT than those with lower incomes (eg, for <$38 000 vs ≥$63 000: OR, 0.41; 95% CI, 0.37-0.45; *P* < .001). Consistent with data presented in Table 2, the odds of receiving SBRT increased with year of diagnosis (2015 vs each year; 2010: OR, 0.36; 95% CI, 0.33-0.40; *P* < .001; 2011: OR, 0.42; 95% CI, 0.38-0.47; *P* < .001; 2012: OR, 0.58; 95% CI, 0.52-0.63; *P* < .001; 2013: OR, 0.77; 95% CI, 0.70-0.85; *P* < .001; 2014: OR, 0.83; 95% CI, 0.76-0.91; *P* < .001). Compared with low-risk disease, the odds of being treated with SBRT decreased with higher-risk disease (OR, 0.27; 95% CI, 0.25-0.30; *P* < .001).

**Table 5.** Multivariable Logistic Regression Model Estimating Stereotactic Body Radiation Therapy Use Among 104 198 Patients^a^

Variable	Odds Ratio (95% CI)	*P* Value	
		Factor Level	Overall
Risk category			
Low	1 [Reference]		<.001
Intermediate			
Favorable	0.86 (0.80-0.93)	<.001	
Unfavorable	0.66 (0.61-0.71)	<.001	
High	0.27 (0.25-0.30)	<.001	
Facility type			
Academic/research program	1 [Reference]		<.001
Community cancer center program	0.22 (0.18-0.25)	<.001	
Comprehensive community cancer program	0.39 (0.36-0.41)	<.001	
Integrated network cancer program	0.46 (0.41-0.51)	<.001	
Facility location			
New England	1 [Reference]		<.001
Mid-Atlantic	3.35 (2.93-3.82)	<.001	
South Atlantic	1.75 (1.52-2.01)	<.001	
Central			
East North	1.03 (0.89-1.19)	.72	
East South	1.95 (1.64-2.32)	<.001	
West North	1.45 (1.22-1.72)	<.001	
West South	1.44 (1.17-1.77)	.001	
Mountain	1.64 (1.34-2.02)	<.001	
Pacific	0.25 (0.20-0.31)	<.001	
Location type			
Metropolitan	1 [Reference]		<.001
Urban	1.27 (1.15-1.41)	<.001	
Rural	1.01 (0.76-1.34)	.95	
Race/ethnicity			
White	1 [Reference]		.02
Black	0.91 (0.84. 0.99)	.02	
Other	0.84 (0.70-1.01)	.06	
Unknown	0.83 (0.65-1.07)	.16	
Primary payer			
Private insurance	1 [Reference]		
Medicaid, Medicare, or other government insurance	0.99 (0.93-1.05)	.70	
Not insured	0.82 (0.62-1.09)	.18	
Unknown	1.68 (1.39-2.04)	<.001	
Median annual income, $			<.001
<38 000	0.41 (0.37-0.45)	<.001	<.001
38 000-47 999	0.42 (0.38-0.46)	<.001	
48 000-62 999	0.53 (0.50-0.58)	<.001	
≥63 000	1 [Reference]		
Charlson-Deyo Comorbidity Index score			
0	1 [Reference]		<.001
1	0.93 (0.85-1.01)	.100	
2	0.61 (0.48-0.79)	<.001	
≥3	0.47 (0.29-0.75)	.002	
Year of diagnosis			
2010	0.36 (0.33-0.40)	<.001	<.001
2011	0.42 (0.38-0.47)	<.001	
2012	0.58 (0.52-0.63)	<.001	
2013	0.77 (0.70-0.85)	<.001	
2014	0.83 (0.76-0.91)	<.001	
2015	1 [Reference]		

^a^ The sample size represents complete cases containing non-missing values for all model factors.

## Discussion

Using a population-based cohort of patients with newly diagnosed prostate cancer, we provide a descriptive analysis of the largest study to date, to our knowledge, exploring trends in the use of SBRT as a definitive treatment modality. Our analyses included 106 926 patients receiving EBRT, of whom 5395 underwent SBRT. The most frequently used total dose for a SBRT treatment course was 36.25 Gy delivered in 5 fractions, which is consistent with prior studies evaluating extreme hypofractionation regimens.^9,10,11,12^ The use of SBRT has more than doubled from 3.1% in 2010 to 7.2% in 2015. These observations are in line with prior studies showing an increase in SBRT use from less than 0.4% in 2004 to 4.0% in 2012.^16,17^ Although this trend persevered among all risk groups, men who underwent SBRT were more likely than those undergoing EBRT to have low-risk disease or favorable intermediate-risk disease, which is consistent with earlier studies.^16,18,19^ Conversely, the number of patients receiving EBRT steadily decreased from 2010 to 2015, which is largely reflective of a consistent decrease in favorable intermediate-risk patients and low-risk patients treated annually during this time. With more low-risk prostate cancer being managed by active surveillance, which is reflected by decreased EBRT in this subset, the observed increasing use of SBRT in this disease subset may be indicative of overtreatment,^20^ patient preference, or increased marketing. Thus, the upward-trending use of both active surveillance and SBRT warrant further clarification regarding when to appropriately implement each option in the low-risk disease setting.

Conversely, although a preference to treat lower-risk disease with SBRT may be reflective of radiation oncologists’ and urologists’ hesitancy to treat higher-risk disease in the context of limited data among this disease subset,^10,21^ there has been a small but steady increase in SBRT use among patients with unfavorable intermediate-risk disease and high-risk disease. Higher-risk patients may be enrolling in clinical trials addressing the efficacy of SBRT in this disease subset (eg, ClincialTrials.gov identifiers NCT02296229, NCT01352598, and NCT01985828). In addition, this observation may be indicative of an initial wavering reluctance to use SBRT among physicians who are regularly using extreme hypofractionation in the treatment of various malignant neoplasms and in the context of emerging long-term treatment outcomes showing favorable comparisons with other radiation modalities.^13^ Nevertheless, larger cohorts and long-term data are required to adequately assess the efficacy of SBRT in higher-risk disease.

As mentioned previously, the proportionally smaller but substantial number of unfavorable intermediate-risk and high-risk patients receiving SBRT and ADT may be attributed to enrollment in clinical trials or to mitigation of burdens due to medical comorbidities or socioeconomic stressors among selected patients. Among the high-risk cohort, 76.6% received ADT, coinciding with a prior study by Chen et al^21^ reporting that 1 of 4 high-risk patients are not treated with ADT. In their analysis, ADT was often omitted in men treated with EBRT and a brachytherapy boost, which was not captured in our data. Androgen deprivation therapy may also be omitted because of patient or physician preference related to its adverse effect profile, which includes sexual dysfunction, osteopenia, metabolic changes, and cardiac toxic effects as well as emerging evidence suggesting that ADT may be associated with depression, cognitive dysfunction,^22^ and the development of Alzheimer disease.^23^ In addition, a study by D’Amico et al^23^ reporting that the benefit of ADT for intermediate-risk and high-risk patients with 7.6 years of follow-up no longer showed a significant benefit after 16 years may give pause to clinicians prescribing ADT because its prostate cancer–specific benefits must be weighed against its adverse effects.

Although national guidelines recommend up-front ADT in conjunction with EBRT for patients with unfavorable intermediate-risk and high-risk disease, routine use is not favored in low-risk prostate cancer and favorable intermediate-risk prostate cancer. Regardless, 9.9% of patients with low-risk disease and 25.7% of patients with favorable intermediate-risk disease underwent ADT and EBRT, with 4.1% of patients with low-risk disease and 10.7% of patients with favorable intermediate-risk disease receiving SBRT and ADT. These findings echo prior reports that up to 10% of the low-risk disease population was prescribed ADT between 2004 and 2012.^24^ Yang and colleagues^25^ attributed ADT use among patients with low-risk disease undergoing EBRT to treatment in nonacademic centers and to the influence of regional practice patterns. In addition, patients with low-risk disease and with favorable intermediate-risk disease may receive ADT to decrease gland size to allow for SBRT. Although the proportion of patients with lower-risk disease treated with ADT is decreasing,^24^ these findings warrant efforts to ensure dissemination of recent data on ADT in the community setting.

Patients undergoing radiotherapy at academic centers were more likely than those treated outside such centers to receive SBRT. This finding is concordant with a prior study by Weiner et al^17^ that showed from 2004 to 2012, SBRT use increased by 7.0% in academic centers, but only by 1.3% in nonacademic institutions. Academic institutions are more likely to obtain and use new treatment modalities and are able to safely evaluate novel management strategies in a clinical trial setting. In addition, they are more likely to be equipped with the necessary technology as well as the physics and physician expertise required for implementation. Prior studies report that a majority of prostate SBRT occurred in the Northeast, which was attributed to a preponderance of academic institutions and facilities with appropriate staffing and technology concentrated in these areas.^16,26^ Conversely, our analysis found that patients in New England and on the Pacific Coast had lower odds than other regions in the United States of receiving SBRT.

Differences in selection of SBRT may reflect patient and physician preference for other treatment regimens or modalities with more robust long-term disease-specific control and safety data compared with that of the existing SBRT literature.^9,10,11,12,13^ Some physicians cite existing data on moderate hypofractionation showing equivalent disease control but with higher grade 2 or worse toxic effects^6^ as a reason to wait on more extensive follow-up before routinely using extreme hypofractionation in their practice. In addition, physician and physicist exposure to hypofractionated and stereotactic treatments in training as well as investing in additional technologies to facilitate effective SBRT delivery, such as fiducial markers, rectal balloons, and implantation of spacers to help protect organs at risk (eg, SpaceOAR Hydrogel), may influence physician selection of SBRT.

Healthier patients were more likely to receive SBRT. Given the slowly progressive nature of prostate cancer, patients with preexisting medical conditions will more likely succumb to those competing comorbidities. Thus, patients with estimated life expectancies of less than 10 years are preferably observed than recommended definitive treatment of their prostatic malignant neoplasm.^2^ Among patients with robust estimated life expectancies, the ensuing prostate cancer management entails interdisciplinary discussions in conjunction with each patient’s preference, intricacies that cannot be fully captured by a database. For example, EBRT may be preferably offered to men who are not deemed surgical candidates owing to their existing comorbidities. By contrast, patients may elect prostatectomy because of preference or to avoid the adverse effects of concurrent ADT with EBRT.

Our analyzed population comprised significantly more white men than black men, the former of which had higher odds of receiving SBRT than other races/ethnicities. Men with higher incomes were more likely to receive SBRT, which was previously discussed in earlier studies and suggests that more affluent men are more apt to seek newer treatment modalities for prostate cancer.^27^ In comparison with those with private insurance, the odds of receiving SBRT was not significantly lower among those with Medicare, Medicaid, or other government-issued insurances, reflecting gradual acceptance of SBRT as a comparably effective treatment option. Halpern and colleagues^16^ reported a median SBRT cost of $27 145, compared with $17 183 for brachytherapy, $37 090 for intensity-modulated RT, and $54 706 for proton beam therapy. Prior studies have noted a decline in brachytherapy use in recent years, which has been attributed to advances in EBRT as well as declining physician training and comfort with the technique.^28^ The relative reimbursements and costs of various equivalent modalities are potential sources of bias in selecting a treatment modality and warrant further investigation. However, as practitioners become more familiar with prostate SBRT and gain confidence in its efficacy as results continue to accumulate from large clinical phase 3 studies, such as the NRG-GU005 trial evaluating SBRT or intensity-modulated RT in stage IIA through IIB prostate cancer, the use of SBRT as a shorter duration, cost-effective, noninvasive RT modality may continue to rise.^29,30^

### Limitations

The findings of this study are subject to the inherent biases of its retrospective nature. These data were derived from the NCDB registry, which comprehensively tabulates an estimated 70% of all new cancer diagnoses,^14,15^ thus limiting the scope of our study to roughly two-thirds of patients diagnosed as having prostate cancer from 2010 to 2015. Despite the thorough data collected by the NCDB, it does not collect information on ADT duration, compliance, and adverse effects. However, because the present study was not intended to report on survival or efficacy, this information would have added significant depth to the analyses but was not vital to our findings. In addition, because the NCDB does not comprehensively capture nuances in RT delivery, future studies are warranted to characterize trends in specific RT planning factors, such as image guidance, use of a rectal balloon, spacers or fiducials, treatment platform (CyberKnife vs linear accelerator), and whether all 5 treatment fractions are delivered on consecutive days. These adjunct technological advances designed to facilitate accurate and safe hypofractionated treatment require physician training and comfort and may also be associated with the frequency at which SBRT is recommended.

## Conclusions

This study found that the use of SBRT as a definitive treatment modality in prostate cancer more than doubled from 2010 to 2015. Androgen deprivation therapy use increased with risk among patients overall, regardless of receiving SBRT. Patients who were treated at an academic center, lived in an urban environment, had higher incomes, were healthier, and were diagnosed as having lower-risk prostate cancer were more likely to receive SBRT. Further investigation is warranted to validate the potential disparities in SBRT implementation to identify and rectify the underlying causes. In addition, the continued use of SBRT will be contingent on ongoing studies reporting long-term outcomes in low-risk prostate cancer and the role of SBRT in higher-risk prostate cancer.

## Notes

Supplement. **eFigure.** Flow Diagram of Patients Included for Analysis
