# Omission in Funding/Support

**DOI:** 10.1001/jamanetworkopen.2020.27727

**Published:** 2020-10-29

In the Original Investigation titled “Trends in the Use of Stereotactic Body Radiotherapy for Treatment of Prostate Cancer in the United States,”^1^ published February 5, 2020, there was an omission in the Funding/Support section in the Article Information. Dr Hu’s research was supported by The Frederick J. and Theresa Dow Wallace Fund of the New York Community Trust. This article has been corrected.^1^
