# Supplementary material for: Trends in the Use of Stereotactic Body Radiotherapy for Treatment of Prostate Cancer in the United States
Source: JAMA Netw Open. 2020 Feb 5;3(2):e1920471. doi: 10.1001/jamanetworkopen.2019.20471 (PMC12068824; doi:10.1001/jamanetworkopen.2019.20471)
Supplement: Supplement. — eFigure. Flow Diagram of Patients Included for Analysis [file jamanetwopen-e1920471-s001.pdf]

Supplementary Online Content

Mahase SS, D'Angelo D, Kang J, Hu JC, Barbieri CE, Nagar H. Trends in the use of stereotactic body radiotherapy for treatment of prostate cancer in the United States. *JAMA Netw Open*. 2020;3(2):e1920471. doi:10.1001/jamanetworkopen.2019.20471

eFigure. Flow Diagram of Patients Included for Analysis

This supplementary material has been provided by the authors to give readers additional information about their work.

© 2020 Mahase SS et al. *JAMA Network Open*.

eFigure. Flow Diagram of Patients Included for Analysis

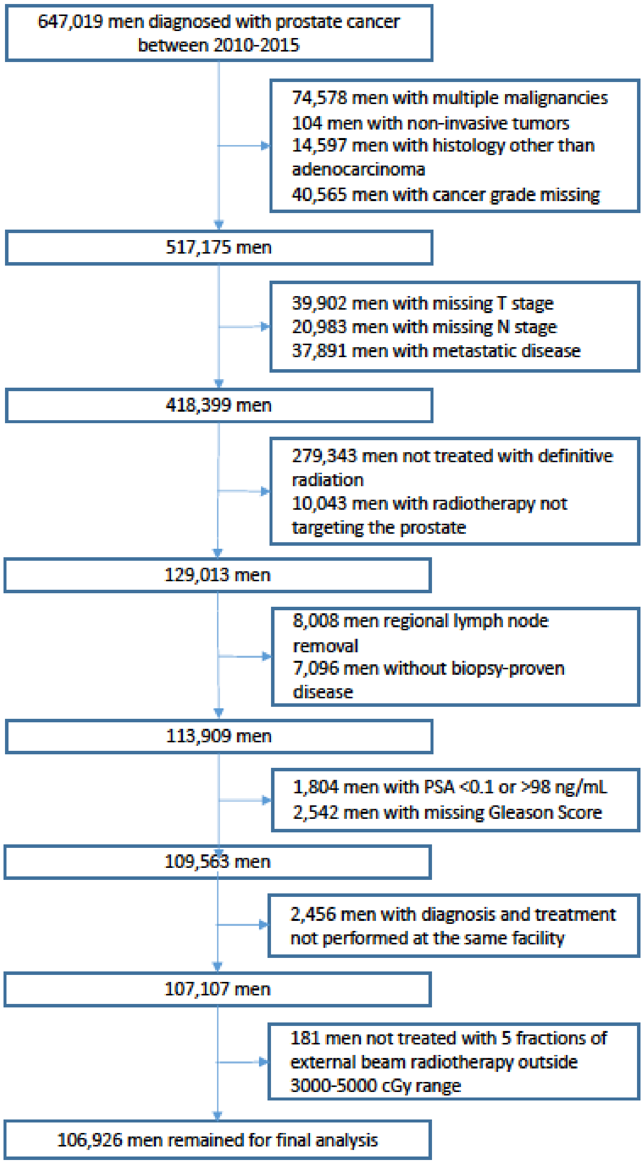

© 2020 Mahase SS et al. *JAMA Network Open.*
